# Using altered auditory feedback to study pitch compensation and adaptation in tonal language speakers

**DOI:** 10.3389/fnhum.2024.1364803

**Published:** 2024-03-19

**Authors:** Ding-lan Tang

**Affiliations:** Human Communication, Development, and Information Sciences, Faculty of Education, The University of Hong Kong, Pokfulam, Hong Kong SAR, China

**Keywords:** altered auditory feedback, pitch perturbation, tonal languages, speech motor control, sensorimotor adaptation

## Abstract

Human speech production is strongly influenced by the auditory feedback it generates. Auditory feedback-what we hear when we speak-enables us to learn and maintain speaking skills and to rapidly correct errors in our speech. Over the last three decades, the real-time altered auditory feedback (AAF) paradigm has gained popularity as a tool to study auditory feedback control during speech production. This method involves changing a speaker’s speech and feeding it back to them in near real time. More than 50% of the world’s population speak tonal languages, in which the pitch or tone used to pronounce a word can change its meaning. This review article aims to offer an overview of the progression of AAF paradigm as a method to study pitch motor control among speakers of tonal languages. Eighteen studies were included in the current mini review and were compared based on their methodologies and results. Overall, findings from these studies provide evidence that tonal language speakers can compensate and adapt when receiving inconsistent and consistent pitch perturbations. Response magnitude and latency are influenced by a range of factors. Moreover, by combining AAF with brain stimulation and neuroimaging techniques, the neural basis of pitch motor control in tonal language speakers has been investigated. To sum up, AAF has been demonstrated to be an emerging tool for studying pitch motor control in speakers of tonal languages.

## 1 Introduction

Speaking is a highly complicated motor behavior. Fluent speech requires not only precise coordination of many muscles, but also integration of auditory and somatosensory feedback. Auditory feedback (i.e., the sounds of our own voice), in particular, is widely acknowledged to be essential for speech production and is a central part of models of speech motor control (Hickok et al., 2011; Guenther, 2016; Parrell et al., 2019). In recent three decades, the real-time altered auditory feedback (AAF) experimental paradigm has provided plentiful evidence that auditory feedback plays a critical role in both online feedback control (e.g., Burnett et al., 1998; Hain et al., 2000) and in updating feedforward control (e.g., Houde and Jordan, 2002; Purcell and Munhall, 2006). Perturbations of auditory feedback introduce prediction errors (i.e., discrepancies) between the predicted and actual auditory feedback. When auditory feedback is perturbed inconsistently, an opposing response (*compensation*) that changes in the opposite direction to the perturbation direction will be elicited to reduce the perceived error within an ongoing production. When auditory feedback is perturbed consistently, speakers can gradually modify their motor plans over multiple successive utterances (*adaptation*) to integrate information from those errors.

By using the AAF paradigm, previous studies have demonstrated pitch compensation and adaptation across various contexts, including singing (Natke et al., 2003), nonspeech vocalization (Larson et al., 2001), and speech production (Jones and Munhall, 2002). Those studies, however, have primarily focused on non-tonal languages (predominantly English), where pitch has a less prominent suprasegmental role. In contrast, around 70% of the world’s languages use changes in pitch (i.e., tones) to alter word meaning, which serve an important linguistic function (Yip, 2002). Such a major difference could potentially affect speakers’ pitch control and determine the extent to which they adjust their pitch in response to pitch perturbations. In recent decades, to investigate the possible influence of a speaker’s entire language system on pitch control, researchers started to examine pitch compensation and adaptation in tonal language speakers. The goal of this mini review is to provide a summary of the progression of altered auditory feedback (AAF) as a method to understand pitch motor control in tonal language speakers. In total, 18 studies (see Table 1) using the AAF paradigm to investigate pitch compensation and adaptation in tonal language speakers were reviewed.

**Table 1:** Summary of the studies included in the review, sorted by study types: pitch compensation (dark yellow), pitch adaptation (dark blue) and neurobiology findings (orange).

**Study**	**Participants**	**Stimuli**	**Perturbation**	**Results**
Jones and Munhall (2000) 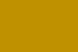	12 native Mandarin speakers	ma1	Inconsistent perturbations (+100 cents and no perturbations)	Participants showed significant compensation (i.e., changed their pitch productions to the opposite direction of the manipulation) with mean response latency of 211 ms.
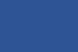	10 native Mandarin speakers		Consistent perturbations (two conditions: +100 cents or – 100 cents)	Adaptation (perturbations applied): participants increased their F0 in both shift-down and shift-up conditions, but the increase was significantly larger in the shift-down condition than in the shift-up condition. Aftereffects (perturbation removed): Participants decreased their F0 in the shift-down condition, while they kept increasing their F0 in the shift-up condition.
Xu et al. (2004) 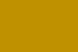	6 native Mandarin (Beijing) speakers	Mandarin phrases (/ma ma/) with different tonal contours, High-High (H-H), High-Rising (H-R), and High-Falling (H-F)	Inconsistent perturbations (±100 cents or no perturbation)	Compared to English speakers, Mandarin speakers showed larger compensatory responses (49–84 cents) when experiencing pitch perturbation to mandarin bitonal sequences.
Liu et al. (2009) 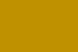	10 native Mandarin speakers	Mandarin sentence (/mao1 mi1 mo1 ma1/, ‘kitty touches mom’) with different intonations (question v.s statement)	Inconsistent perturbations (±100 cents or no perturbation)	Compared to meaningless disyllabic Mandarin phrases, smaller compensatory responses (20 ~ 30 cents) were elicited during Mandarin sentence productions. The timing of perturbations affected response magnitudes (question intonation only): response magnitudes (16 cents) were significantly decreased for the 340 ms (after vocal onset) condition compared to the 160 (26 cents) or 240 (23 cents) conditions.
Ning et al. (2014) 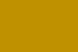	10 native English speakers 10 L2 learners of Mandarin 9 native Mandarin speakers	Vowel /a/	Inconsistent perturbations (±50 or ±100 cents)	No significant difference between groups were found in response amplitude or latency. Compared native English speakers and L2 Mandarin learners, the F0 contours of Mandarin speakers were least affected by the amplitude and direction of pitch perturbations.
Ning et al. (2015) 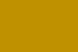	10 native English speakers 10 L2 learners of Mandarin 10 native Mandarin speakers 6 trained vocalists with English as their native language	/ma1 ma1/, /ma1 ma2/, /ma1 ma4/, and the vowel /a/	Inconsistent perturbations (±200 cents)	During productions of sustained vowel, both native Mandarin speakers and trained vocalists showed significantly reduced response amplitudes in comparison to native English speakers. During productions of Mandarin bitonal sequences, only native Mandarin speakers, but not trained vocalists, exhibited significantly smaller magnitude deviation in comparison to native English speakers.
Liu et al. (2010) 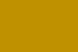	18 native Mandarin speakers 18 native Cantonese speakers	vowel /u/	Inconsistent perturbations (±50, ±100, ±200, and ±500 cents)	Cantonese speakers exhibited smaller compensatory responses than Mandarin speakers when the stimulus magnitude varied from 200 to 500 cents.
Ning (2019) 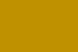	26 native Taiwanese Southern Min (TSM) speakers	TSM words: /un55-ding33/ (HM); /un33-too33/ (MM); /un11-tong33/ (LM)	Inconsistent perturbations (±250 or ±150 cents)	Larger and faster compensatory responses were elicited when the shifted pitch overlaps with another level tone: when the H level tone of the HM word was downshifted by 250 cents to the M level, and when the L level tone of the LM word was upshifted by 150 cents to the M level. No such effect was found for the MM word.
Ning (2022b) 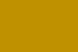	24 native TSM speakers	TSM words with different tonal contours: Flat--HH (tsau51-im55), MM(kau55-uann33), LL (pan33-an11) Rising--MH (oo55-im55), LM(ti33-an55), LH (mia33-un33) Falling--MH (ho51-un33), LM(koo51-i11), LH (oo55-am11)	Inconsistent perturbations (±250 or ±150 cents)	Pitch perturbation to the flat contour resulted in smaller pitch compensation compared to the rising and falling contours. Downshifting the rising contour or upshifting the falling contour (i.e., when the shifted pitch overlapped with the M tone) did not result in larger pitch compensation. The timing of perturbations affected response magnitudes: pitch compensation at 100 ms was smaller than at 400 ms for the falling contour, but not for the flat and rising contours.
Jones and Munhall (2005) 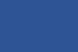	9 native Mandarin speakers	/ma1/ and /ma2/	Consistent perturbations (+100 cents)	Adaptation: participants lowered their F0 in response to consistent F0 shift-up; adaptation for one tone category (tone 1) can be generalized to the production of another tone category (tone 2). Aftereffects: participants increased their F0.
Feng et al. (2018) 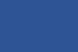	9 native Mandarin speakers	/ma1/, /ma2/ /ma3/, /ma4/	Consistent perturbations (−100 cents on tone 1 only; −100 cents on tone 1 and +100 cents on tone 3)	Adaptation: Significant adaptation was observed for the shifted tone 1 and unshifted tone 4, while no significant adaptation was found for unshifted tone 2 and tone 3; When receiving simultaneous tone 1 (− 100 cents) and tone 3 (+ 100 cents) perturbations, no significant tone 1 adaptation was observed, and tone 3 adaptation occurred only if tone 2 was also produced. Aftereffects: in the shift-down condition on tone 1, participants held their F0 at approximately the same level as observed during the baseline phase and throughout the perturbation phases.
Ning (2018) 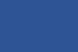	12 native Mandarin speakers 7 L2 Mandarin beginners 7 L2 Mandarin advanced learners	vowel /a/ and /ma1/	Consistent perturbations (+100 cents or −100 cents)	Adaptation: L2 beginners showed greater adaption (70 cents) than L2 advanced learners (61 cents) and native Mandarin speakers (56 cents), while no significant difference in adaptation was found between L2 advanced learners and native Mandarin speakers. Aftereffects: All three groups exhibited significant aftereffects in the downward shift of /ma1/, whereas no significant aftereffects were observed in the upward shift.
Ning (2020) 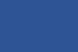	15 Mandarin non-vocalists 15 Mandarin vocalists	vowel /a/ /ma1/ and /ma2/	Consistent perturbations (+100 cents or −100 cents)	Adaptation: vocalists showed less adaptation than non-vocalists, Aftereffects: aftereffects appeared to (no statistical evidence) be present in the responses of all speakers to the downward shift of /ma1/ and /ma2/, but not the upward shift.
Liu et al. (2020) 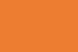	21 native Mandarin speakers	vowel /u/	Inconsistent perturbations (+200 or +500 cents)	Inhibitory cTBS over DLPFC elicited larger compensatory responses and smaller event-related potential (ERP) P2 responses, compared to sham stimulation.
Chang et al. (2023) 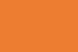	17 native Mandarin speakers		Inconsistent perturbations (±50 or ± 200 cents)	Active tDCS over DLPFC led to significantly faster but smaller compensatory responses than sham stimulation.
Dai et al. (2022) 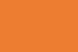	20 native Mandarin speakers		Inconsistent perturbations (−50 or −200 cents)	Inhibitory cTBS over the left SMA led to decreased pitch compensations.
Li et al. (2023) 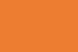	20 native Mandarin speakers		Inconsistent perturbations (±200 cents)	HD-tACS (6 or 70 Hz) over the left IFG resulted in larger but slower compensatory responses, paralleled with larger ERP P2 responses than sham HD-tACS.
Li et al. (2022) 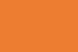	24 native Mandarin speakers			Inhibitory cTBS over both the left and right SMG led to smaller pitch compensations and smaller ERP P2 responses.
Liu et al. (2023) 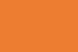	23 native Mandarin speakers		Inconsistent perturbations (−50 or − 200 cents)	Inhibitory cTBS over the right, but not left pSTG led to smaller pitch compensations and smaller ERP N1 and larger P2 responses.

IFG, inferior frontal gyrus; DLPFC, dorsolateral prefrontal cortex; STG, superior temporal gyrus; SMA, supplementary motor area; cTMS, continuous theta burst stimulation; tDCS, transcranial direct current stimulation; tACS, transcranial alternating current stimulation.

## 2 Pitch compensation

Previous studies in non-tonal language speakers have shown that participants lowered their pitch when F0 was shifted up and raised their pitch when it was shifted down (e.g., Hain et al., 2000; Jones and Munhall, 2000). Jones and Munhall (2002) first investigated pitch compensation in tonal language speakers. They found that similar to non-tonal language speakers, Mandarin speakers changed their pitch productions to the opposite direction of the manipulation when inconsistent pitch perturbations (+ 100 cents or no perturbation) were introduced to their auditory feedback during production of Mandarin word /ma/ with a high level tone (tone 1, ‘mother’). Mean response latency was 211 ms, which was longer than non-tonal language speakers producing sustained vowels (114 ms, Hain et al., 2000; 130 ms, Larson et al., 2001). The response magnitudes, however, were not reported in Jones and Munhall (2002). In a later study, compensatory responses to pitch perturbation (±100 cents or no perturbation) were examined in Mandarin speakers when they produced disyllabic Mandarin phrases (/ma ma/) with different tonal contours, High-High (H-H), High-Rising (H-R), and High-Falling (H-F) (Xu et al., 2004). They found that Mandarin speakers had larger compensatory responses (49–84 cents) when experiencing pitch perturbation to mandarin bitonal sequences, than English speakers’ compensatory responses to pitch perturbation when producing the sustained vowel /a/ (30 ~ 40 cents, Hain et al., 2000; Larson et al., 2001). The results seem to suggest that pitch feedback control in tonal languages is more sensitive than that in non-tonal languages. The same research group further investigated the compensatory responses to pitch perturbation in Mandarin speakers when producing Mandarin sentence (/mao1 mi1 mo1 ma1/, ‘kitty touches mom’) with different intonation patterns (statement v.s question) (Liu et al., 2009). Compared to meaningless disyllabic Mandarin phrases, generally smaller compensatory responses (20 ~ 30 cents) were elicited during Mandarin sentence productions.

Ning et al. (2014) conducted an initial study where they directly compared pitch compensation between speakers with and without tonal language experience. Three groups of participants (native English speakers who were never exposed to tonal languages, L2 learners of Mandarin whose native language was English, native Mandarin speakers) vocalizing the same vowel /a/ while receiving F0 perturbation with different magnitudes (±50 and ± 100 cents). No significant difference between groups were found in response amplitude or latency. In addition, the authors modeled the entire F0 contours using generalized additive models and found that F0 contours of the native English speakers and L2 learners were affected by perturbation magnitude, but Mandarin speakers were not. The authors thus suggested that instead of increasing pitch-control sensitivity, tonal language experience may contribute to the development of a more stable vocal control. In a later study, the same group of researchers further investigated whether compensatory responses induced by pitch perturbation were affected by language or vocal training experiences (Ning et al., 2015). The compensatory responses of four groups of participants (the same three language groups as in their previous study and trained vocalists with English as their native language) during production of Mandarin bitonal sequences /ma1 ma1/, /ma1 ma2/, /ma1 ma4/, and the vowel /a/ were examined. In contrast to previous findings, both native Mandarin speakers and trained vocalists showed significantly reduced response amplitudes when producing sustained vowel compared to native English speakers. During productions of Mandarin bitonal sequences, only native Mandarin speakers, but not trained vocalists, exhibited significantly smaller magnitude deviation in comparison to native English speakers. The authors concluded that tonal language speakers have more robust or stable pitch control during both linguistic (Mandarin bitonal sequences) and nonlinguistic (sustained vowel) productions and are thus less affected by auditory feedback perturbations. Liu et al. (2010) showed that pitch motor control even differed across different tonal languages. Cantonese speakers exhibited significantly smaller responses than Mandarin speakers when receiving relatively large pitch perturbations (±200, or ± 500 cents).

More recently, Ning (2019, 2022b) conducted two studies in speakers of Taiwanese Southern Min to investigate the impact of pitch contour on pitch compensation. The author proposed that larger and faster compensatory responses would occur when the shifted pitch overlaps with another level tone. The results showed that when the H level tone of the HM word (/un^55^-ding^33^/) was downshifted by 250 cents to the M level, and when the L level tone of the LM word (/un^11^-tong^33^/) was upshifted by 150 cents to the M level, larger compensatory responses were elicited. However, no such effect was found for the MM word (/un^33^-too^33^/) (Ning, 2019). Specifically, upshifting the M level tone of the MM word to the H level or downshifting the M level tone of the MM word to the L level did not result in larger pitch compensation. In the later study, the author further examined how different pitch contours would affect pitch compensation and proposed that pitch perturbation to the flat contour (e.g., MM), where speakers could maintain stability more easily, would result in smaller pitch compensation compared to the rising (e.g., LM) and falling (e.g., HM) contours. The results support the author’s hypothesis, indicating that flat pitch contour exhibited a greater resistance to pitch perturbation compared to the rising or falling pitch contours. However, unlike the previous study, downshifting the rising contour or upshifting the falling contour (i.e., when the shifted pitch overlapped with the M tone) did not result in larger pitch compensation (Ning, 2022b).

### 2.1 Interim summary

In sum, by using the AAF paradigm, existing work has shown that tonal language speakers are able to compensate for pitch perturbation during production of sustained vowel, nonsense and meaningful speech. Response magnitudes were relatively smaller during meaningful speech in comparison to nonsense speech. In terms of the influence of the two different language systems (tonal vs. non-tonal languages) on pitch compensation, we still cannot reach a consensus. Early study found larger compensatory responses in Mandarin speakers than that in English speakers, suggesting tonal language speakers are more sensitive to pitch perturbation (Xu et al., 2004). However, different stimuli were used in previous tonal and non-tonal language studies. Ning and colleagues directly compared pitch compensation between tonal and non-tonal language speakers using the same stimuli and perturbations. The results showed that tonal language speakers have attenuated rather than enhanced compensatory responses when experiencing pitch perturbation, suggesting that tonal language speakers may have a more entrenched pitch control system, making them less susceptible to auditory perturbations (Ning et al., 2015). However, it should be noted that in their earlier study, no significant difference was found in response amplitude between tonal and non-tonal language speakers (Ning et al., 2014). The mixed results may stem from large variability among participants and relatively small sample sizes in previous studies. A fairly large portion of people in auditory perturbation studies demonstrated minimal compensation (10–40%, Lametti et al., 2012) or even exhibited following responses (i.e., production altered in the same direction as the perturbation) (23%, MacDonald et al., 2011). A recent study showed that following responses can reach 35–55% in tonal language speakers when experiencing pitch perturbation (Ning, 2022a). Even though such large variability is found among participants, a sample size of 10 (or less) per group is typical in the reviewed studies (see Table 1). To gain more clarity on this significant question, future research with a larger sample size is necessary.

## 3 Pitch adaptation and aftereffect

### 3.1 Sensorimotor adaptation

With consistent auditory feedback perturbation, speakers can exhibit sensorimotor adaptation, in which they gradually modify and update their feedforward commands of the motoric plan over multiple successive utterances. Pitch adaptation in tonal language speakers was first examined in the study of Jones and Munhall (2002), in which they found Mandarin speakers adapted for pitch perturbations during Mandarin word production (/ma1/). Specifically, participants increased their F0 in both shift-down and shift-up conditions (±100 cents) but the increase was significantly larger in the shift-down condition than in the shift-up condition. In a later study, the same researchers showed that Mandarin speakers lowered their F0 in response to consistent F0 shift-up. Moreover, they found that adaptation for one tone category (tone 1) can be generalized to the production of another tone category (tone 2) (Jones and Munhall, 2005). A more recent study investigated simultaneous adaptation to pitch perturbation in Mandarin speakers, in which single and simultaneous perturbations to different tone categories were applied during the production of Mandarin words (Feng et al., 2018). When receiving single perturbation (−100 cents on tone 1), Mandarin speakers exhibited significant adaptation on the shifted tone. Such adaptation generalized to production of tone 4 (high falling tone), but not tone 2 (mid rising tone) nor tone 3 (low dipping tone). When receiving simultaneous tone 1 (shift-down) and tone 3 (shift-up) perturbations, no significant tone 1 adaptation was observed, and tone 3 adaptation occurred only if tone 2 was also produced.

Ning (2018, 2020) examined the influences of tonal language and vocal training experiences on pitch adaptation. In the study of Ning (2018), pitch adaptation during productions of sustained /a/ and /ma1/ in L2 Mandarin beginners, L2 Mandarin advanced learners, and native Mandarin speakers were examined. L2 beginners showed greater adaption (70 cents) than L2 advanced learners (61 cents) and native Mandarin speakers (56 cents). This is consistent with the author’s previous findings in pitch compensation, suggesting greater exposure to tonal languages would lead to a more stable pitch control system which is less affected by auditory feedback perturbations. In the author’s later study, pitch adaptation in trained vocalists and non-vocalists whose native language is Mandarin were compared (Ning, 2020). Overall, when it comes to pitch shift during the production of /a/, /ma1/, or /ma2/, vocalists exhibited less adaptation than non-vocalists, indicating that similar to tonal language experience, vocal training also contributes to a more entrenched pitch control system.

### 3.2 Aftereffects

Adaptive changes persist even after feedback is returned to normal (*aftereffects*), which is considered as a clear sign of changes in feedforward control (Jones and Munhall, 2000; Lametti et al., 2018). However, mixed results have been reported regarding the aftereffects during pitch adaptation.

An early study examining aftereffects in English speakers found that English speakers decreased their F0 in the shift-down condition, while they kept increasing their F0 in the shift-up condition, when the feedback returned to normal (Jones and Munhall, 2000). Similar pattern of aftereffects has been observed in Mandarin speakers during Mandarin word production (ma1) (Jones and Munhall, 2002). Moreover, the aftereffects can be generalized from one tone category to another tone category (Jones and Munhall, 2005). Feng et al. (2018), however, reported a different pattern of aftereffects in their native Mandarin speakers’ tone production. Specifically, in the shift-down condition on tone 1, after feedback was restored to normal, speakers did not decrease their F0. Instead, they held their F0 at approximately the same level as observed during the baseline phase and throughout the perturbation phases.

In more recent studies of Ning (2018, 2020), aftereffects were found to vary across stimuli and shift direction. In Ning (2018), all three groups with different tonal language experience (see 3.1) exhibited significant aftereffects in the downward shift of /ma1/, whereas no significant aftereffects were observed in the upward shift. The author suggested that reducing F0 for the upward shift in the high flat tone (ma1) might require less correction effort compared to increasing it for the downward shift. Consequently, in the case of the upward shift, participants’ F0 quickly returned to baseline levels once the auditory perturbation was removed. No significant aftereffect during production of /a/ was found neither (except for L2 advanced learners’ upward shift /a/), which contradicts previous findings (Jones and Keough, 2008; Keough and Jones, 2009). In the later study, although statistical evidence was not provided, aftereffects appeared to be present in the responses of all speakers to the downward shift of /ma1/ and /ma2/, but not the upward shift (Ning, 2020).

### 3.3 Interim summary

The reviewed studies have shown that tonal language speakers are able to adapt when consistent perturbations in pitch feedback are introduced. However, there is mixed evidence regarding the aftereffects. The discrepancies may be attributed to large participants variability and methodological differences, including the number of produced tone categories (a single tone vs. all four tone categories) and production stimuli (sustained vowel vs. word production). Further research is needed to clarify these points.

## 4 Neurobiology findings

By combining AAF paradigm with brain stimulation and neuroimaging techniques, a series of studies have been conducted by Liu’s group to investigate the neural basis of pitch control in tonal language speakers (e.g., Liu et al., 2020, 2023; Li et al., 2022). They have demonstrated the causal role of left dorsolateral prefrontal cortex (DLPFC) in pitch compensation by showing that inhibitory continuous theta burst stimulation (cTBS) over DLPFC elicited larger compensatory responses and smaller event-related potential (ERP) P2 responses in Mandarin speakers experiencing pitch perturbation (+200 or +500 cents), compared to sham stimulation (Liu et al., 2020). In contrast, transcranial direct current stimulation (tDCS) over DLPFC led to significantly faster but smaller compensatory responses than sham stimulation (Chang et al., 2023). The authors suggested that DLPFC is involved in top-down inhibitory control over vocal motor behavior. Altering the function of left DLPFC, either by disrupting or enhancing it, can result in impairment or enhancement of this top–down inhibitory control, which regulates the extent to which feedback perturbations influence speech production. The involvement of left inferior frontal gyrus (IFG) and supplementary motor cortex (SMA) in pitch compensation in Mandarin speakers have also been demonstrated in studies of Dai et al. (2022) and Li et al. (2023), in which enhanced or reduced compensatory responses to pitch perturbation were observed after transcranial alternating current stimulation (tACS) over left IFG or cTBS over left SMA, respectively.

Previous research has indicated that the posterior superior temporal gyrus (pSTG) plays a critical role in both speech perception and production (Okada and Hickok, 2006). The dual-stream model suggests that the pSTG, predominantly in the left hemisphere (Hickok and Poeppel, 2007), is responsible for encoding auditory-phonological representations, while the Directions Into Velocities of Articulators (DIVA) model proposes that both the left and right pSTG are involved in auditory feedback control (Guenther et al., 2006). However, it is important to note that these models are primarily based on findings from English speakers. In a recent study, a right-lateralized contribution of the pSTG to pitch feedback control has been found in tonal language speakers (Liu et al., 2023). More specifically, cTBS over right, but not left pSTG resulted in reduced compensations for pitch perturbations accompanied by smaller N1 and larger P2 responses in Mandarin speakers. In contrast, both the left and right supramarginal gyrus (SMG) has been found to causally contribute to pitch compensation in Mandarin speakers (Li et al., 2022).

### 4.1 Interim summary

Overall, by combining AAF paradigm with brain stimulation and neuroimaging techniques, several brain areas, including PFC, pSTG, SMG have been demonstrated to play an important role in pitch compensation in tonal language speakers. It is worth noting that in those studies, the response magnitudes were generally smaller (~15 cents) compared to the magnitudes observed in behavioral studies (~30 cents). It remains unclear whether the differences can be attributed to the presence of neuroimaging/brain stimulation techniques or the use of different stimuli (/u/ v.s /a/ or /ma/). In addition, we did not find any studies that investigate the neural basis of pitch adaptation in tonal language speakers.

## 5 Conclusion

By using AAF paradigm, pitch compensation and adaptation has been demonstrated in tonal language speakers. The compensation and adaptation are under the influence of various factors, such as the type of perturbation stimuli, the speaker’s language background, and their vocal training. However, the exact influence of tonal language experience on pitch control still remains unclear.

## Author contributions

D-LT: Writing – original draft, Writing – review & editing, Writing – original draft, Writing – review & editing.
